# The TIR-Domain Containing Adaptor TRAM Is Required for TLR7 Mediated RANTES Production

**DOI:** 10.1371/journal.pone.0107141

**Published:** 2014-09-11

**Authors:** Enda Shevlin, Sinéad M. Miggin

**Affiliations:** Institute of Immunology, Department of Biology, National University of Ireland Maynooth, Maynooth, Co. Kildare, Ireland; SRI International, United States of America

## Abstract

Toll-like receptor 7 (TLR7) plays a vital role in the immune response to ssRNA viruses such as human rhinovirus (HRV) and Influenza, against which there are currently no treatments or vaccines with long term efficacy available. Clearly, a more comprehensive understanding of the TLR7 signaling axis will contribute to its molecular targeting. TRIF related adaptor molecule (TRAM) plays a vital role in TLR4 signaling by recruiting TRIF to TLR4, followed by endosomal trafficking of the complex and initiation of IRF3 dependent type I interferon production as well as NF-κB dependent pro-inflammatory cytokine production. Towards understanding the molecular mechanisms that regulate TLR7 functionality, we found that TRAM^−/−^ murine macrophages exhibited a transcriptional and translational impairment in TLR7 mediated RANTES, but not TNFα, production. Suppression of TRAM expression in human macrophages also resulted in an impairment in TLR7 mediated CCL5 and IFN-β, but not TNFα, gene induction. Furthermore, suppression of endogenous human TRAM expression in human macrophages significantly impaired RV16 induced CCL5 and IFNβ, but not TNFα gene induction. Additionally, TRAM-G2A dose-dependently inhibited TLR7 mediated activation of CCL5, IFNβ and IFNα reporter genes. TLR7-mediated phosphorylation and nuclear translocation of IRF3 was impaired in TRAM^−/−^ cells. Finally, co-immunoprecipitation studies indicated that TRAM physically interacts with MyD88 upon TLR7 stimulation, but not under basal conditions. Our results clearly demonstrate that TRAM plays a, hitherto unappreciated, role in TLR7 signaling through a novel signaling axis containing, but not limited to, MyD88, TRAM and IRF3 towards the activation of anti-viral immunity.

## Introduction

TLRs function by recognizing conserved structural motifs, or pathogen associated molecular patterns (PAMPs) derived from infectious organisms and initiating an intracellular signaling cascade which in turn brings about the appropriate innate and adaptive immune response. Many aspects of their expression, localisation, activation and downstream signaling are tightly regulated by an ever expanding panel of both positive and negative regulators [1]. One immediate method of regulation is the recruitment of various Toll/Interleukin 1 receptor (TIR) domain containing adaptor proteins – of which there are five [2]. MyD88 is required for all TLR signaling, except TLR3, and causes pro-inflammatory cytokine production via activation of NF-κB and the mitogen activated protein kinases (MAPKs). TRIF is utilised by TLR3 and TLR4 causing interferon regulatory factor (IRF) and NF-κB induction leading to the production of both inflammatory cytokines and type I Interferon (IFN). Mal, also known as TIRAP, functions as a sorting adaptor, recruiting MyD88 to TLR2 and TLR4. Similar to Mal, TRAM functions to recruit TRIF to TLR4 [2]. TRAM and TLR4 have been shown to traffic between the plasma and endosomal membranes from where they initiate pro-inflammatory and anti-viral signaling [3]. Further downstream signaling complexes are designated as either MyD88 dependent or TRIF dependent (MyD88 independent). Finally, SARM negatively regulates TLR3 and TLR4 signaling by inhibiting TRIF recruitment [4]. More recently, novel roles for the TLR adaptors have been delineated. For example, MyD88 and Mal have been shown to negatively regulate TLR3 signaling [5], [6]. Also, TRIF has been implicated in both the positive and negative regulation of TLR5 signaling [7], [8]. TRAM has been shown to be required for maximal IL-18R signaling [9].

The endosomal TLRs, TLR7, TLR8 and TLR9 are part of an evolutionary cluster believed to have arisen via an X-linked duplication event approximately 150 million years ago [10]. TLR7 was initially shown to sense synthetic antiviral imidazoquinoline derivatives such as imiquimod and resiquimod (R-848) [11], [12] and, thereafter, was shown to detect single stranded RNA (ssRNA) derived from RNA viruses such as Influenza A and human immunodeficiency virus (HIV). Notably, studies have also shown that TLR7 can sense RNA derived from bacteria [13]. Expression of TLR7 is somewhat restricted to tissues including the lung and brain [10]. In resting cells, TLR7 is sequestered in the endoplasmic reticulum and rapidly trafficks to the endolysosome via UNC93B1 upon infection [14]. Infecting virus particles are enveloped and internalised to the endolysosome where they encounter TLR7. Upon activation, MyD88 binds to constitutively expressed IRF7 leading to the formation of a multiprotein complex including IRAK1, IRAK4, TRAF6, TRAF3 and IKKα, which in turn leads to the phosphorylation of IRF7 and subsequent translocation to the nucleus [14]. Similar to the trafficking of MyD88 to the endosome following TLR7 engagement [15], TRAM has also been shown to traffick to the endosome upon TLR4 engagement [3]. Given this and the newly ascribed roles for the TLR adaptor proteins in TLR signaling [6], [16], we sought to explore whether TRAM plays a functional role in TLR7 signaling. Using murine immortalised bone marrow derived macrophages (iBMDMs) generated from TRAM-deficient mice, we found that TLR7 mediated RANTES production was suppressed when compared to wild type mice. Moreover, suppression of human TRAM expression using RNA interference in human macrophages resulted in a decrease in *CCL5*, *IFN-β*, but not *TNFα*, expression following ssRNA virus human rhinovirus 16 (HRV-16) infection. Mechanistically, we show that TRAM myriostoylation, but not Mal, is required for TLR7 mediated activation of CCL5, IFN-α and IFN-β reporter gene activity through a mechanism that involves the phosphorylation and nuclear translocation of IRF3. To our knowledge, our study shows for the first time that the TIR-adaptor domain containing protein TRAM is required for maximal TLR7 mediated RANTES and IFN-β production.

## Materials and Methods

### Cell Culture and Reagents

HEK293-TLR7 cells were a generous gift from Professor Stefan Bauer, University of Marburg. Wild type and TRAM^−/−^ iBMDM cells were provided by Professor Luke O’Neill, Trinity College Dublin. All cells were grown in DMEM, high glucose (Sigma) supplemented with 10% fetal calf serum, 1% penicillin-streptomycin, 1% sodium pyruvate and maintained at 37°C in a humidified atmosphere of 5% CO_2_. G418 (250 µg/ml) was added to maintain the HEK293-TLR7 cells. Lipopolysaccharide from *E. coli*, Serotype EH100(Ra) (Alexis), high molecular weight Poly(I:C), CLO97 and R848 were purchased from Invivogen. The siRNA oligonucleotides were synthesised by Sigma-Aldrich using the following sequences: TRAM sense, 5′-UUGGAUAUUUAUAAUGGGUTT-3′, and antisense, 5′-ACCCAUUAUAAAUAUCCAATT-3′. Negative ‘scrambled’ control sequences were 5′-UAUAAUUCAAUCACACAACTT-3′ (sense) and 5′-GUUGUGUGAUUGAAUUAUATT-3′ (antisense). Human rhinovirus 16 (HRV16) was a gift from Professor Steve Goodbourn, University of London.

### Expression Vectors/Recombinant Plasmids

The reporter gene constructs IFN-β-luciferase, IFN-α-luciferase and RANTES-luciferase were as previously described [16]. The Flag-TRIF was as described [17]. The plasmids pcDNA3: MyD88-cmyc, Flag-TRAM and TRAM-G2A were generously provided by Professor Luke O’Neill (Trinity College Dublin).

### Sources of macrophages

TRAM^−/−^ mice were constructed as described [18]. WT and TRAM mice were on a C57BL/6 background. All mice were confirmed as being homozygous mutants by PCR genotyping of DNA. All the animal protocols used in this study were approved by the Ethical Committee at the National University of Ireland, Maynooth and in accordance with the Animals (Scientific Procedures) Act, 1986, UK. iBMDMs were generated as previously described [19].

### First Strand cDNA synthesis

Total RNA was isolated from all types of cells using TRI REAGENT according to the manufacturer’s instructions (Sigma). Thereafter, total RNA was converted to first strand cDNA as described (25). Briefly, 1 µg RNA was incubated with 2 µl random hexamer primers (500 µg/ml) at 70°C for 5 min. Thereafter, the additional reaction components were added in the following order: 5 µl of 5×RT buffer (Fisher), 1.3 µl of 10 mM dNTP (New England Biolabs), 0.5 µl RNase Inhibitor (Fisher), 0.5 µl MMLV Reverse transcriptase (Fisher) and nuclease-free water to a total volume of 25 µl. The tubes were incubated at 37°C for 40 min and at 42°C for 40 min followed by heating to 80°C for 5 min.

### Real-time PCR

Total cDNA was used as starting material for real-time RT-PCR quantitation with SYBR Green JumpStar *Taq* ReadyMix (Sigma) on a real-time PCR system (Light Cycler 480; Roche). For the amplification of the specific genes the following primers were used; *hIFN-α*, forward, GAAATACTTCCAAAGAATCACTCT and reverse, GATCTCATGATTTCTGCTCTGACA; *mTNFα,* forward, CATCTTCTCAAAATTCGAGTGACAA and reverse, TGGGAGTAGACAAGGTACAACCC; *hTNFα*, forward, CCCAGGGACCTCTCTCTAATCA and reverse, AGCTGCCCCTCAGCTTGAG, *mCCL5*, forward, GGAGATGAGCTAGGATAGAGGG and reverse TGCCCATTTTCCCAGGACCG; *hCCL5,* forward, TGTGGTAGAATCTGGGCCCTTCAA and reverse, TGCCTGTTTCTGCTTGCTCTTGTC. For each mRNA quantification, the housekeeping gene *GAPDH* was used as a reference point using the following primers, *mGAPDH* forward, GCACAGTCAAGGCCGAGAAT, and reverse, GCCTTCTCCATGGTGGTGAA; *hGAPDH* forward, TCGACAGTCAGCCGCATCTTCTTT, and reverse, ACCAAATCCGTTGACTCCGACCTT. It was confirmed that the expression of GAPDH was not affected by the various treatments. Real-time PCR data were analysed using 2^–ΔΔCT^ method as described [20].

### siRNA transfection

THP1 cells were differentiated using Phorbol 12-myristate 13-acetate (PMA; 40 nM) for 48 hr. The medium was then replaced with fresh RPMI. After 4 hr, cells were transfected with siRNA to target the suppression of TRAM. Briefly, for each well in a 12 well plate, 200 nM of siRNA was transfected into 1.4×10^6^ cells in 1 ml of medium using 4 µl of lipofectamine (Life Technologies) per well. After 48 hr, the efficiency of TRAM knockdown was assessed by Real Time-PCR using human TRAM forward TCCACAGTGATGCCTACTGATGCT and reverse primers ATGCAGATGAGAGGTGGACCCATT and GAPDH forward AGCTTGCTGGTGAAAAGGAC and reverse primers TTATAGTCAAGGGCATATCC.

### Transfection and co-immunoprecipitation

HEK293-TLR7 cells (1.4×10^6^ cells/well; 6-well plate) were allowed to reach 70% confluency upon which cells were co-transfected with 3 µg Flag-TRAM and 3 µg EV or 3 µg Flag-TRAM and 3 µg Myc-MyD88 using Lipofectamine 2000. After 24 hr, cells were either left unstimulated or stimulated with CLO97 (5 µg/ml) for 15, 30 and 60 min as indicated. Thereafter, cells were lysed in 200 µl low stringency buffer (LSB) (50 mM HEPES, pH 7.5, 150 mM NaCl, 2 mM EDTA pH 7.6, 1% NP-40, 0.5% sodium deoxycholate supplemented with 1 mM PMSF, 1 mM DTT, 1 mM NaVO_3_, 5 mM EGTA and protease inhibitor cocktail). Cellular debris was removed by centrifugation and then 20 µl of the remaining whole cell lysate (WCL) was removed, mixed with an equal volume of 5x Laemmli loading buffer, boiled for 10 min and stored at –20°C until required for WCL analysis. Next, 1 µg of anti-Flag M2 monoclonal antibody (Sigma, F3165) was added to the remaining cell lysates followed by incubation overnight at 4°C with gentle shaking. Next, 25 µl Protein A/G beads (Santa Cruz) were added followed by incubation overnight at 4°C with gentle shaking. Samples were then washed 4 times with unsupplemented LSB followed by the addition of 50 µl of 5x Laemmli loading buffer and boiling for 10 min. Samples were subjected to SDS-PAGE gel electrophoresis and immunoblot analysis using the indicated antibodies.

### Reporter Assays

HEK293-TLR7 cells (4×10^5^ cells/well; 96 well plate) were transfected with 60 ng/well luciferase reporter gene plasmid for CCL5, IFN-β and IFN-α as previously described (9) and co-transfected with the expression vector pcDNA3:TRAM-G2A using Lipofectamine 2000 as described by the manufacturer (Life Technologies). In all cases, 40 ng/well of phRL-TK reporter gene was co-transfected to normalize data for transfection efficiency. After 24 hr, cells were stimulated with CLO97 (5 µg/ml) as indicated. Thereafter, cell lysates were prepared and reporter gene activity was measured using the Dual Luciferase Assay system (Promega) as described (27). Data was expressed as the mean fold induction ± S.D. relative to control levels, for a representative experiment from a minimum of three separate experiments, each performed in triplicate.

### Cytokine analysis

iBMDMs (1×10^6^ cells per well) were stimulated with various TLR ligands. After 24 hr, the cell supernatants were removed and analysed for TNFα and RANTES cytokine release as indicated by the manufacturer (Peprotech).

### Extraction of Cellular Nuclear Fraction

iBMDMs were stimulated with R848 (1 µg/ml), Poly(I:C) (25 µg/ml) or LPS (100 ng/ml) for 0–2 hr. After ligand stimulations, the cells were collected and nuclear extracts were prepared using the nuclear Extraction Kit as described by the manufacturer (Cayman Chemical). Thereafter, the nuclear fraction was subjected to immunoblot analysis using anti-IRF3 (Santa Cruz, sc-9082) and anti-Lamin A/C (Cell Signaling, 2032s) antibodies.

### pIRF3 and IκBα immunoblot analysis

Cells were stimulated with Poly(I:C) (25 µg/ml), LPS (100 ng/ml) and R848 (1 µg/ml) as described and whole cell lysates were subjected to SDS-PAGE followed by immunoblot analysis with an IκBα (Cell Signaling Technology), an anti-phospho-IRF3 (Cell Signaling Technology), an anti-IRF3 (Santa Cruz) and anti-β-actin (Sigma) antibodies.

### Data analyses

Statistical analysis was carried out using the unpaired Student’s t test using Graphpad Prism 5 programme. P-values of less than or equal to 0.05 were considered to indicate a statistically significant difference where * indicated p<0.05 and ** indicates p<0.005.

## Results

### TRAM is required for TLR7 mediated RANTES production

**Figure 1:**
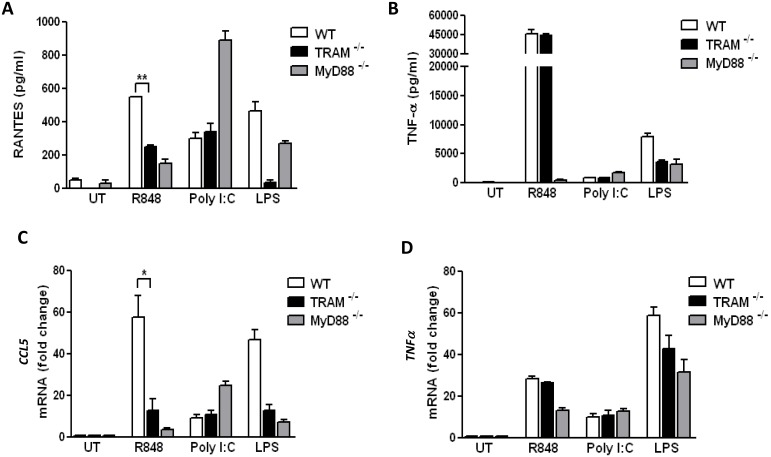
R848 mediated RANTES, but not TNF-α production, is significantly decreased in TRAM^−/−^ iBMDMs. (A, B) Immortalised iBMDMs from WT, TRAM**^−/−^**, and MyD88**^−/−^** mice were treated with R848 (1 µg/ml), Poly (I:C) (25 µg/ml) or LPS (100 ng/ml) for 16 hr as indicated. Thereafter, RANTES (A), and TNF-α (B) were measured by ELISA as described in Materials and Methods. Results presented are from a single experiment and are representative of at least three independent experiments performed in triplicate (mean ± SE). (C, D) Immortalised iBMDMs from WT, TRAM**^−/−^** and MyD88**^−/−^** mice were treated with R848 (1 µg/ml), Poly(I:C) (25 µg/ml), or LPS (100 ng/ml) for 5 hr. Thereafter, total RNA was isolated, converted to first-strand cDNA, and used as a template for quantitative real-time RT-PCR as described in Materials and Methods. Quantitative real-time PCR was used to assay the expression levels of *CCL5* (C), and *TNF-α* (D). Results presented are the mean values from at least three independent experiments performed in duplicate (mean ± SE) where GAPDH was used to normalize all samples.

Previous studies conducted by our group have shown novel roles for the TIR-domain containing adaptors MyD88 and Mal/TIRAP in TLR signaling [5], [6]. Specifically, we have shown that MyD88 and Mal play a negative role in TLR3 mediated type I IFN production, via inhibition of IRF3 and IRF7 respectively [5], [6]. Given these findings, we sought to explore whether an understudied TLR adaptor protein, TRAM, may have a hitherto unappreciated role in TLR signaling, distinct from it’s know role in TLR4 signaling. To investigate the role of TRAM in TLR7 signaling, we opted to use three alternative TLR7 stimuli, namely R848, CLO97 and a physiologically relevant virus, namely HRV16. Initially, we measured TLR7-mediated RANTES and TNFα production by ELISA in TRAM^−/−^ and WT cells and demonstrate that levels of RANTES were suppressed in TRAM^−/−^ iBMDMs when compared to WT iBMDMs following stimulation with the TLR7 ligand, R848 (Fig. 1A). In contrast, comparable levels of R848 mediated TNFα secretion were evident in WT and TRAM^−/−^ iBMDMs (Fig. 1B). Moreover, comparable RANTES and TNFα production was evident in TRAM^−/−^ iBMDMs when compared to WT cells following stimulation with Poly(I:C), but not LPS (Fig. 1A, B). As expected, impaired levels of RANTES and TNFα secretion were evident in MyD88^−/−^ iBMDMs compared to WT iBMDMs following stimulation with R848 (Fig. 1A). Next, the role of TRAM in the transcriptional regulation of TLR7 mediated RANTES and TNFα was explored. Correlating with ELISA data, real-time PCR data revealed that R848 mediated CCL5 induction was significantly decreased in TRAM^−/−^ iBMDMs when compared to WT cells (Fig. 1C). As a control, we show that R848 mediated CCL5 and TNFα induction was suppressed in MyD88^−/−^ cells compared to WT iBMDMs (Fig. 1C, D). As expected, comparable CCL5 and TNFα induction was evident in TRAM^−/−^ iBMDMs when compared to WT cells following stimulation with Poly(I:C), but not LPS (Fig. 1C, D). Together, these data suggest that TRAM is required for TLR7 mediated CCL5 gene induction.

**Figure 2:**
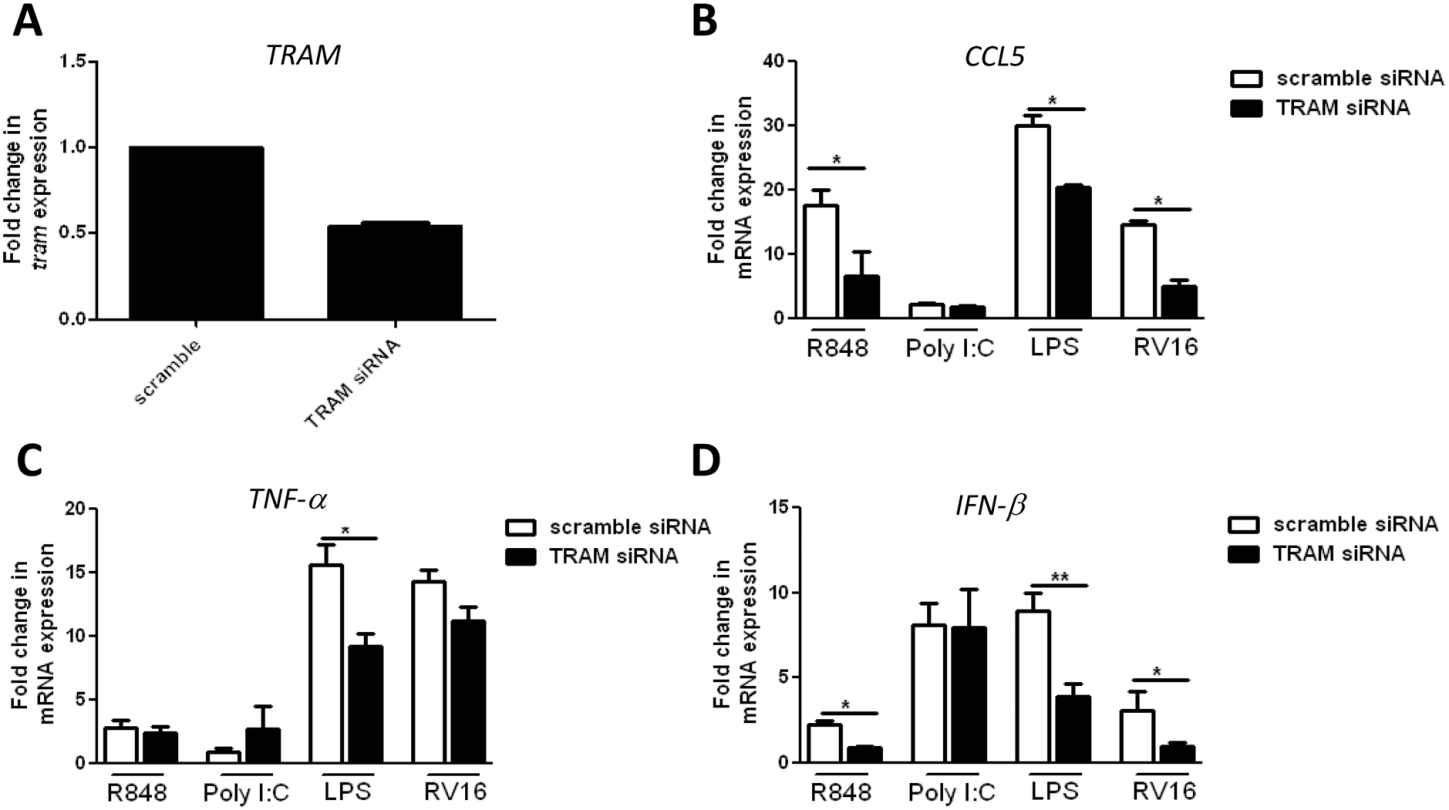
Suppression of endogenous human TRAM expression decreases R848 mediated *CCL5* and *IFN-β*, but not *TNF-α* expression. (A–D) THP-1 cells were differentiated with PMA for 48 hr followed by transfection with either scrambled control or TRAM siRNA to target the suppression of TRAM. After 60 hr, cells were stimulated with R848 (3 µg/ml), Poly(I:C) (25 µg/ml), LPS (1 µg/ml) or Rhinovirus-16 (MOI: 5 for 80 hr) for 8 hr, unless otherwise stated. Next, total RNA was isolated, converted to first-strand cDNA and used as a template for quantitative real-time RT-PCR as described in Materials and Methods to assay the expression levels of *TRAM* (A), *CCL5* (B), *TNF-α* (C) or *IFN-β* (D). The data presented is the mean ± SE of two independent experiments each performed in duplicate (mean ± SE).

To preclude the possibility of species dependent differences in TRAM functionality in the context of TLR7 signaling, TRAM expression was suppressed in human macrophages using siRNA technology (Fig. 2A) and thereafter, TLR driven cytokine production was assessed. Specifically, human monocytic THP1 cells were differentiated into macrophages using PMA, followed by suppression of TRAM expression using TRAM-specific siRNA or control non-specific scramble siRNA for 60 hr and stimulation with R848, LPS or Poly I:C for 8 hr. Correlating with data generated using TRAM^−/−^ murine iBMDMs, suppression of TRAM expression in human macrophages resulted in a significant decrease in R848 and LPS, but not Poly(I:C) mediated CCL5 induction when compared to cells transfected with the scrambled control siRNA (Fig. 2B). In contrast, comparable R848 and Poly(I:C) mediated TNFα induction was evident in WT and TRAM^−/−^ cells (Fig. 2C). As TLR7 mediated induction of CCL5, but not TNFα mRNA was impaired in TRAM deficient cells, this indicated that TRAM was leveraging TLR7 signaling not via NF-κB, but perhaps via the IRF pathway and thus may also affect transcription of the IFN-β gene [6], [21]. Hence, the role of TRAM in the transcriptional regulation of IFN-β was also examined wherein it was found that suppression of TRAM expression resulted in a significant decrease in R848 and LPS, but not Poly(I:C) mediated IFN-β induction when compared to control cells (Fig. 2D).

To investigate the physiological role of TRAM in the modulation of virally induced CCL5 and IFN-β induction, TRAM expression was suppressed in differentiated THP1 cells and cells were then treated with the ssRNA virus [16], HRV16 followed by assessment of CCL5, TNFα and IFN-β gene induction. It was found that suppression of TRAM using siRNA technology suppressed RV16 induced CCL5 and IFN-β induction without significantly affecting TNFα gene induction when compared to controls (Fig. 2A–D). Taken together, these data indicate that TRAM is required for TLR7-mediated and virally induced human CCL5 and IFN-β gene induction.

### TRAM is required for TLR7-induced CCL5, IFN-α and IFN-β reporter gene activity

**Figure 3:**
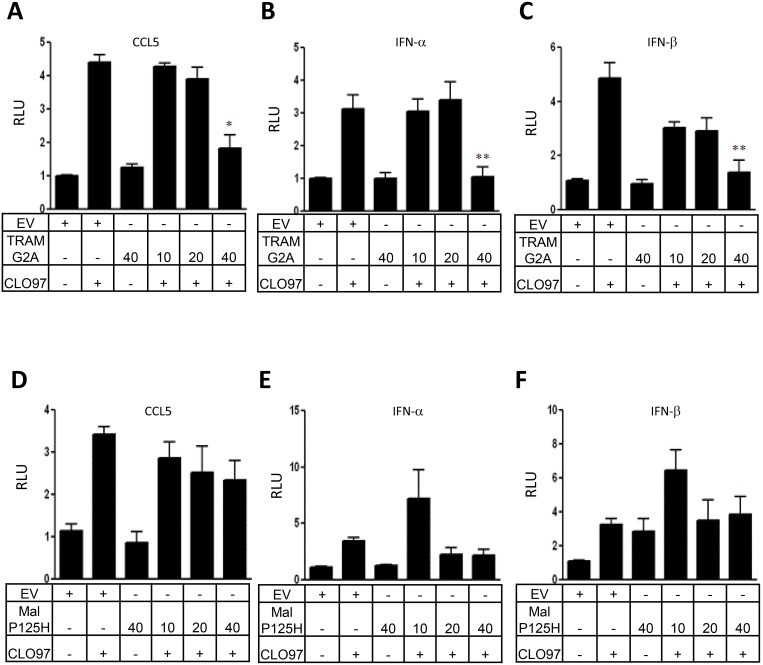
Dominant negative TRAM (TRAM-G2A) negatively regulates TLR7-mediated CCL5, IFN-α and IFN-β reporter gene activity. (A–F) HEK293-TLR7 cells were cotransfected with vectors encoding either a luciferase reporter gene for CCL5 (A, D), IFN-α (B, E) or IFN-β (C, F) and either empty vector (pcDNA3; 40 ng) or increasing amounts of an expression vector encoding TRAM-G2A (10, 20, 40 ng) or Mal-P125H (10, 20, 40 ng) as indicated. After 24 hr, cells were stimulated with CLO97 (5 µg/ml). A total of 40 ng/well phRL-TK (TK-Renilla-luciferase) reporter gene was co-transfected simultaneously to normalize data for transfection efficiency After 24 hr, cell lysates were harvested and assessed for luciferase reporter gene activity. In all cases, results are expressed as mean ± SE for triplicate determinants of single experiments. Each experiment was performed a minimum of 3 times with a representative chosen for graphical purposes. Statistical analysis was performed using unpaired student t-test.

Following the correct folding of TLR7 within the endoplasmic reticulum (ER) lumen, it is trafficked through the Golgi by the conventional secretory pathway and then routed to endosomes where it can encounter its ligand [22], [23]. TRAM has previously been shown to localise to the plasma membrane and Golgi in resting cells but can also traffic independently of TLR4 to endosome membranes via a bipartite sorting motif [3]. TRAM is regulated by myristoylation, which is required for the adaptor molecule to be localized within plasma membrane and mutation of the myristoylation motif abolishes its functional activity [3], [24]. With this in mind, we examined whether expression of a myristoylation defective TRAM protein, TRAM-G2A, affected TLR7 mediated transcription factor activation. To this end, HEK293 cells stably transfected with TLR7 (HEK293-TLR7) to render them TLR7 ligand responsive were transiently transfected with the CCL5, IFN-α and IFN-β reporter gene constructs and increasing amounts of TRAM-G2A. After 24 hr, cells were stimulated with the TLR7 ligand, CLO97. We found that transfection of HEK293-TLR7 cells with TRAM-G2A dose-dependently inhibited CLO97 induced activation of the CCL5, IFN-α and IFN-β reporter genes (Fig. 3A–C). As a control, we show that dominant negative Mal-P125H did not significantly affect CCL5, IFN-α and IFN-β reporter gene activity in HEK293-TLR7 cells (Figure 3D–F). Taken together, these data show that TRAM, but not Mal, is required for optimal TLR7 mediated CCL5 and IFN-β reporter gene activity.

### TRAM is required for TLR7 mediated IRF3 activation and interacts with MyD88 in a TLR7 dependent manner

**Figure 4:**
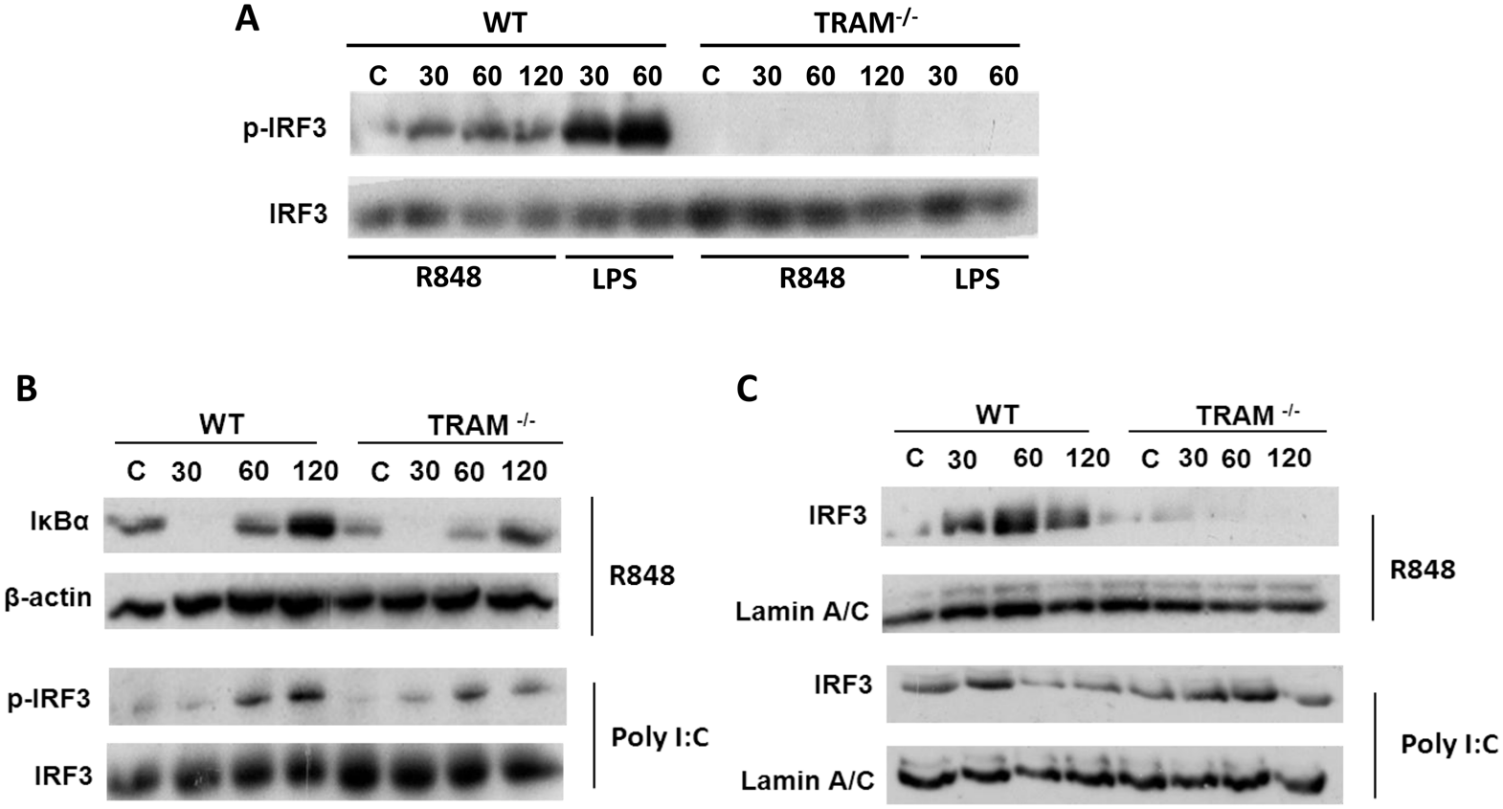
R848 mediated IRF3 activation, but not IκBα degradation, is abolished in TRAM^−/−^ iBMDMs. (A, B) WT and TRAM**^−/−^** iBMDMs were stimulated with R848 (1 µg/ml) Poly(I:C) (25 µg/ml) or LPS (100 ng/ml) for 30, 60 and 120 min. Next, the cell lysates were harvested and phospho-IRF3 signaling was assessed by immunoblot analysis with total protein serving as a loading control. Additionally, WT and TRAM**^−/−^** iBMDMs were stimulated with R848 (1 µg/ml) and immunoblot analysis was performed using an anti IκBα antibody with β-Actin serving as a loading control. The results presented are representative of at least three independent experiments. (C) Alternatively, WT and TRAM**^−/−^** iBMDMs were stimulated with R848 (1 µg/ml) and Poly(I:C) (25 µg/ml) for 0–120 min as indicated. Next, the nuclear fractions were generated and levels of nuclear IRF3 were assessed by immunoblot analysis with Lamin A/C serving as loading control. The results presented are representative of at least three independent experiments, except for Poly(I:C), which is representative of 2 independent experiments.

Given that our data suggests a role for TRAM in the transcriptional regulation of TLR7 mediated RANTES and IFN-β, but not TNFα, we hypothesised that this may reflect an underlying specificity in terms of transcription factor utilisation via a TLR7-TRAM signaling axis. As TRAM has previously been shown to be required for TLR4 mediated IRF3 activation in macrophages [3], we examined whether TLR7 engagement may also result in the activation of IRF3 in macrophages and, more importantly, whether this effect is mediated in a TRAM-dependent manner. To test this, iBMDMs from WT and TRAM^−/−^ mice were stimulated with the TLR7/8 ligand R848 for 30, 60 and 120 min. Thereafter, immunoblot analysis was performed using an anti-phospho IRF3 antibody to assess IRF3 phosphorylation status with increased phosphorylation indicating enhanced IRF3 activity. The TLR7/8 ligand R848 induced the phosphorylation of IRF3 in a time dependent manner (Fig. 4A). In contrast, R848 dependent phosphorylation of IRF3 was not evident in TRAM^−/−^ iBMDMs. To support our hypothesis that TRAM is required for R848 mediated IRF3, but not NF-κB, activation, we examined R848 mediated IκBα degradation, a marker of NF-κB activity, in WT and TRAM^−/−^ iBMDMs. Comparable R848 mediated IκBα degradation was evident in WT and TRAM^−/−^ iBMDMs suggesting that TRAM is not required for TLR7 mediated NF-κB activity (Fig. 4B). As additional controls, we show comparable Poly(I:C) mediated phosphorylation of IRF3 in WT and TRAM^−/−^ iBMDMs (Fig. 4B) and as expected, LPS mediated IRF3 phosphorylation was abolished in WT and TRAM^−/−^ iBMDMs (Fig. 4A). As IRF3 phosphorylation in required for its nuclear translocation, we examined whether loss of TRAM similarly affected TLR7 mediated nuclear translocation of IRF3. Correlating with the IRF3 phosphorylation data, stimulation of WT iBMDMs with R848 mediated increases in the level of nuclear IRF3, as evident 30–60 min post stimulation (Fig. 4C). In contrast, R848 did not induce the nuclear translocation of IRF3 in TRAM^−/−^ iBMDMs. As a control, we demonstrate comparable Poly(I:C) mediated IRF3 nuclear translocation in WT and TRAM^−/−^ iBMDMs (Fig. 4C). Taken together, these data strongly suggest that IRF3 is activated following TLR7 engagement and that TRAM is required for this process.

**Figure 5:**
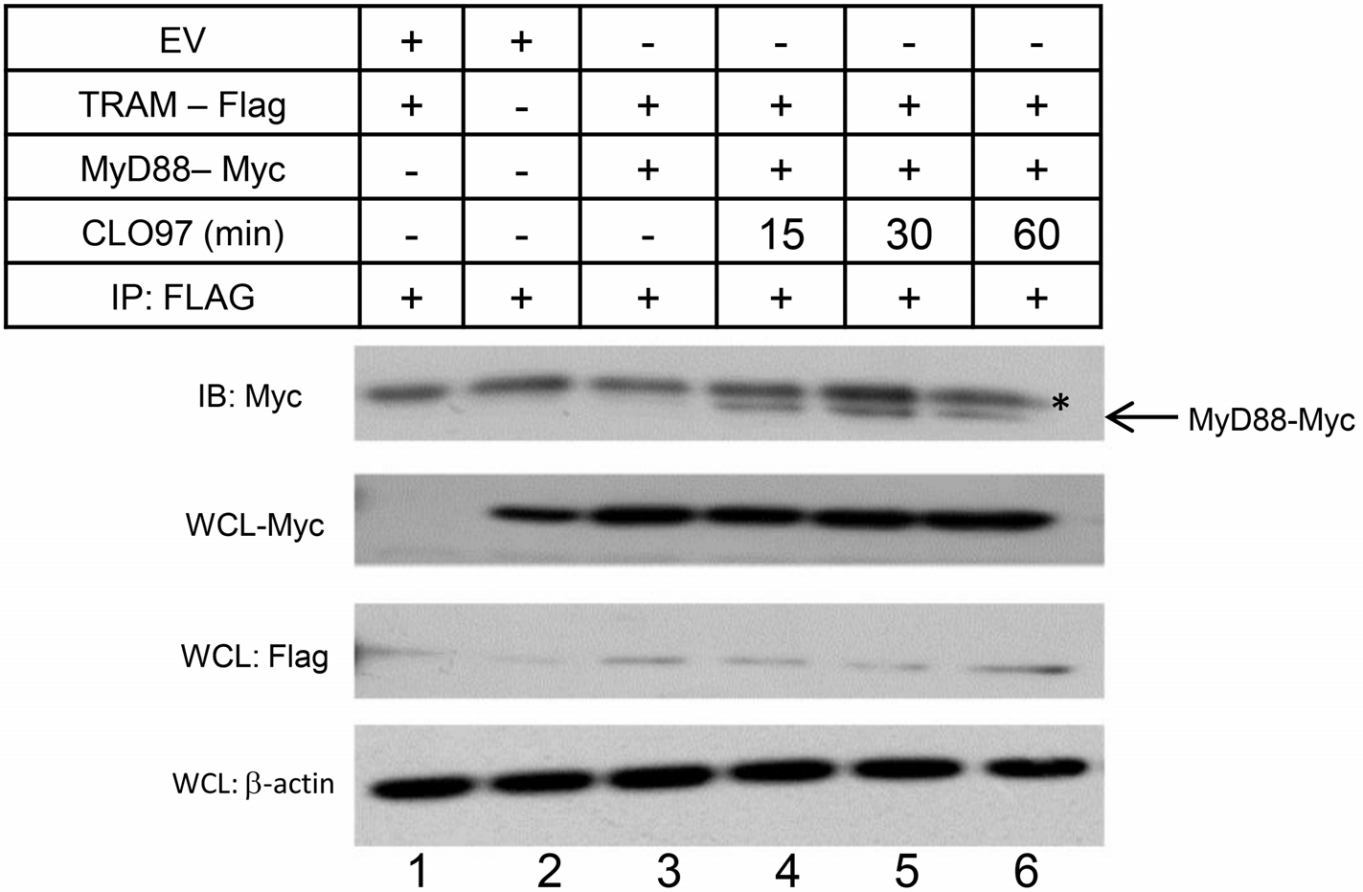
TLR7 mediated association of TRAM with MyD88. HEK293-TLR7 cells were seeded into a 6-well plate at a density of 1.4×10^6^ cells/well and incubated for approximately 24 hr at 37°C. Cells were then co-transfected with vectors encoding TRAM-Flag, MyD88-myc or empty vector (EV). After 24 hr, cells were either left unstimulated or stimulated with CLO97 (5 µg/ml) for 15, 30 and 60 min as indicated. Next, cell lysates were prepared as described in Materials and Methods. An aliquot (20 µl) was removed for whole cell lysate (WCL) analysis. Thereafter, the lysates were subjected to immunoprecipitation (IP) using an anti-Flag antibody followed by western blot analysis using the indicated antibodies. Results are representative of three independent experiments.

A recent study demonstrated that TRAM can act as a linker molecule between MyD88 and the IL-18 receptor (IL-18R), allowing IL-18 signaling to be transduced in a manner similar to how TRAM interlinks between TLR4 and TRIF [9], [25]. Using overexpression studies, the group demonstrated a ligand-independent interaction between TRAM and MyD88 with dissociation occurring following activation of the IL-18R with exogenous IL-18 [9]. Also, a separate study demonstrated that TRAM does not directly interact with TLR7 in resting cells but does interact with TLR4 [26]. Given these findings, it is plausible to speculate that TRAM may participate in TLR7 signaling though a mechanism that involves an interaction with MyD88, rather than TLR7. To test this hypothesis, co-immunoprecipitation studies were performed wherein HEK293-TLR7 cells were co-transfected with Flag-tagged TRAM and Myc-tagged MyD88 for 24 hr, followed by stimulation with CLO97 for 0–60 min. Here, we demonstrate that whilst MyD88 and TRAM do not interact in the absence of ligand stimulation, an interaction between TRAM and MyD88 was evident following TLR7 engagement using CLO97 (Fig. 5, compare lane 3 with lanes 4–6).

## Discussion

The TIR-domain containing adaptor protein TRAM has until recently, been associated exclusively with TLR4 signaling, acting as a linker molecule to bridge TLR4 with TRIF, towards activation of MyD88 independent anti-viral signaling [1]. More recently, a novel role has been attributed to TRAM in IL-18 signaling wherein TRAM was shown to act as a linker molecule between MyD88 and IL-18R thus enabling downstream inflammatory cytokine production [9]. Also, a separate study demonstrated that TRAM^−/−^ mice exhibited a greater susceptibility to TLR2-driven *Francisella tularensis* infection when compared to WT mice indicating a possible, yet to be dissected, role for TRAM in TLR2 signaling [27]. Currently, MyD88 is the only TIR-domain containing adaptor protein purported to modulate TLR7 signaling [1], [12]. Previous indications of a role for TRAM in TLR7/8 signalling have suggested that TRAM may be involved in driving TLR7 mediated NF-κB activation [25]. Another study, using macrophages from TRAM-deficient mice, indicated that TRAM did not play a role in TLR7 mediated production of the NF-κB controlled cytokines TNFα and IL-6 [18]. Therefore, while a role for TRAM in TLR7/8 signalling has previously been alluded to, it remains controversial.

Given that previous studies undertaken by our group have delineated new roles for the TLR adaptor molecules TIRAP and MyD88 in TLR signaling and that TRAM remains a hitherto, relatively uncharacterised molecule in terms of its functionality in innate immunity, we opted to explore whether TRAM affected TLR signaling pathways, distinct from its known role in TLR4 and TLR2 signaling. To this end, we provide data that convincingly attributes a role for TRAM in TLR7 mediated production of RANTES and IFN-β, but not TNFα. Given that our study demonstrates that TLR7 mediated induction of IRF-dependent type I IFN and CCL5 genes are modulated by TRAM, it is plausible to speculate that TRAM may affect the functionality of IRFs. Regarding the role of IRFs in antiviral immunity, studies have suggested that IRF3 and IRF7 are the master regulators of type I IFN production [28], [29], wherein both molecules may act in concert to orchestrate a protective anti-viral immune response [29], [30]. Further, studies have also revealed that IRF5 and IRF7 are involved in TLR7 signaling [31], [32], though cell-type dependent disparities exist in terms of their relative contribution to anti-viral immunity [28]. Given these studies, coupled to the unavailability of phospho-IRF5 antibodies, we opted to measure TLR7 mediated IRF3 activity by immunoblot analysis using phospho-specific IRF3 antibodies as previously described by our group [16]. In contrast to a previous study which showed that IRF3 was not involved in TLR7 signaling [32], our study clearly demonstrates a time dependent induction of IRF3 phosphorylation following TLR7 engagement. Our study also demonstrates that TLR7, but not TLR3, mediated phosphorylation of IRF3 is impaired in TRAM^−/−^ cells when compared to WT iBMDMs. To support this hypothesis, we tested whether TRAM affected the concomitant nuclear translocation of IRF3. We show that levels of TLR7 mediated nuclear translocation of IRF3 are impaired in TRAM^−/−^ iBMDMs when compared to WT cells. In contrast, comparable levels of nuclear IRF3 were evident following stimulation of WT and TRAM^−/−^ cells with the TLR3 ligand, Poly(I:C). Taken together, these data suggested to us that IRF3 is involved in TLR7 signaling through a mechanism that involves TRAM.

Next, we sought to further investigate the mechanism utilised by TRAM to modulate the TLR7 mediated IRF3 functionality. Whilst it is known that MyD88 interacts with both TLR7 and TRAM [9], [25], and that TRAM does not directly interact with TLR7 in unstimulated cells [26], the dynamics of the association between TRAM and MyD88 in the context of TLR7 are unknown. In contrast to IL-18R signaling, our co-immunoprecipitation study revealed that the interaction between MyD88 and TRAM is facilitated by receptor engagement though TLR7. Regarding the co-immunoprecipitation itself, the level of MyD88 that is detected following TLR7 engagement varies commensurate with the level of a non-specific band at 40 kDa. Whilst we are unable to state that the strength of the interaction between MyD88 and TRAM is modulated over time, we can conclusively state that MyD88 and TRAM do interact in a manner that is dependent on TLR7 activation. The complete absence of Myc-MyD88 in lane 3 (unstimulated) and the presence of Myc-MyD88 in lanes 4–6 (CLO97 stimulated) is, we believe, evidence that TRAM and MyD88 do interact, and cannot be explained by a slight increase in the intensity of the non-specific 40 kDa band in the lane 5 when compared to the other lanes. Thus, we believe that MyD88 and TRAM interact through a mechanism that requires TLR7 engagement. Regarding localization, studies have shown that TRAM is located at the plasma membrane and in the cytoplasm of resting cells and trafficks to the endosome upon TLR4 activation [3], [33]. Pathogen challenge may facilitate the endosomal localization of TRAM and concomitant interaction with the ‘TLR7 signalsome’. MyD88 also trafficks from the cytoplasm of resting cells to endosomal compartments upon TLR7 and TLR9 activation [34], [35]. As TRAM has been shown to interact with IRF3 [25], it is plausible to speculate that TRAM may facilitate the recruitment of IRF3 to the endosomally localized TLR7:MyD88 signaling complex. Further, the contrasting role of TRAM in IL-18 receptor and TLR7 signaling highlights the importance of delineating the role played by signaling molecules in varying biological milieu.

The majority of our current knowledge regarding the role played by TLR7 in anti-viral signaling emanates from studies conducted using plasmacytoid dendritic cells (pDCs) as they secrete comparably higher levels of type I IFN relative to macrophages and conventional dendritic cells [36]–[38]. TLR7 mediated production of both type I IFN and inflammatory cytokines requires MyD88 and indeed IRAK4 and TRAF6. Downstream, the signaling bifurcates wherein type I IFN secretion has a requirement for IRAK-1 while the IKK complex (IKKβ, γ) is required to drive NF-κB activation and concomitant proinflammatory cytokine production [37], [38]. Comparatively, limited studies have focused on understanding the dynamics of TLR7 mediated IFN signaling in macrophages. To our knowledge, our study is the first to describe a hitherto unappreciated role for TRAM in anti-viral cytokine induction mediated by the TLR7 pathway. Contra to an absolute requirement for MyD88 in TLR7 signaling, it is notable that the loss of TRAM does not abolish TLR7 signaling [12]. Interestingly, preliminary results from our laboratory suggest that TRIF, but not Mal, is also involved in TLR7 mediated RANTES, but not TNFα, production (data not shown). It would therefore be of interest to further explore the role of TRIF, a TLR adaptor linked with anti-viral immunity, in TLR7 signaling. In addition, studies from our laboratory also suggest that TRAM, but not Mal, is involved in TLR9 mediated RANTES, but not TNFα, production (data not shown). Whilst TRAM is required for TLR4 mediated anti-viral signaling, TLR4 mediated MyD88-dependent signaling remains intact in TRAM deficient cells. Also, IL-18 signaling can still occur in TRAM deficient cells, albeit impaired [9]. Accordingly, therapeutic targeting of TRAM may offer a strategy towards the suppression of antiviral signaling whilst preserving MyD88 dependent signaling. In conclusion, our study provides TRAM as a novel modulator of TLR7 mediated IRF3 activation serving as an additional element to tailor the host immune response to viral infection that mediates their effects through TLR7.
